# Generators of Uric Acid in the Organism

**Published:** 1875-11

**Authors:** 


### GENERATORS OF URIC ACID IN THE ORGANISM.

Nearly all the nitrogen contained in the albumen, which passed into the blood by the alimentary canal, leaves the organism either under the form of urea (men and carnivorous animals) or under the form of uric acid (birds and some serpents.)

Since the discovery of uric acid in the urine of birds by Fourcroy and Vauquelin, till 1848, the question about the generators of uric acid in the organism was not touched. At this time, Strahl and Liberkuhn found uric acid in the blood of cats, whose kidneys were dissected, but neither in the blood taken from frogs and dogs, from which the kidneys were extirpated, nor in the normal blood of birds, did they find uric acid.

There exists a physiological difference in the process of excretion between urea and uric acid, first noticed by Galvani. He observed that uric acid was precipitated in different organs of birds, under the form of a white powder, after tying there ureters. Observing this phenomenon, we are enabled to trace the excretory process itself, while urea, when accumulated in the organism, becomes not visible. These facts make it evident that the question about the generators of urea (or its physiological equivalent, uric acid) may be solved only by the chemical way. This was pursued by Zalesky. He found a precipitation of uric acid after the extirpation of the kidneys in snakes, on those places which were occupied by the kidneys. Had he only tied the ureters, the kidneys continued to excrete uric acid, which, after some hours, was to be observed in many organs in a precipitated state. As in the normal blood of birds there could not be detected, by him, any uric acid, and as the precipitation of uric acid first appeared in the kidneys after tying the ureters, Zalesky concluded that the kidneys produce the uric acid. But Meissner did not give credit to his views, and undertook to make new experiments relating to this subject. He detected uric acid in larger quantities of blood taken from fowls, than Zalesky had found. He then searched for uric acid in the different organs, but found in most of them scarcely traces of it. Only the liver contained a larger amount, and he concluded from this fact that this organ is, in a normal state, the principal generator of uric acid.

At last the microscopical examinations of Chrzonszczewsky must be mentioned. He intended to trace up the origin of lymph vessels, by the aid of the precipitations of uric acid. Having tied the ureters of fowls, he found that the precipitations appeared first in the kidneys, which were followed, ten to twelve hours afterwards, by such in the connective tissue cells, contrary to Zalesky's observations. A few hours later the lymph vessels began to fill up, by which process the connection between these and the connective tissue cells could be observed. At a later hour precipitations appeared on the serous membranes, while they disappeared in the connective tissue cells. The related facts induced Chrzonszczewsky to consider the connective tissue cells, especially their nuclei, around which (next to the kidneys) the precipitations appear first, as the true generators of uric acid.

The views of the different investigators on the generators of uric acid deviate from each other so widely; nay, as facts obtained by one observer were shown to be unreliable by another, the author undertook a series of experiments with the view to solve the question. His results were as follows: He found uric acid in the normal blood of fowls fed on meat, but not of those fed on barley or bread. In the latter he thinks it might exist in too small a quantity to be detected. When he tied the ureters of pigeons, he found post-mortem, a number of precipitations upon the serous membranes, in the straight canals of the kidneys and in the lymph vessels. They were first noticed in the lymph vessels, and in some cases, at the same time, in the connective tissue cells, somewhat later around the capillaries and smaller arteries in the serous membranes. A disappearance of the precipitate from the connective tissue cells (Chrzonszczewsky) was not noticed. It is evident that the observations related do not support Zalesky's assertion, that the precipitations are spread from the kidneys as a center. The places where the precipitates were observed, and the order in which they followed, point to the blood system as containing, at least, the material for the formation of the acid. After tying the ureters the precipitate appears first in those places where the least hindrance to its excretion is found, i. e., kidneys, lymph vessels, serous membranes. This is further confirmed by the disposition of the acid around the blood vessels. Thus it is established that the material for precipitation is taken from the blood. We do not know in what relation the uric acid appears in the blood. The author thinks that perhaps a neutral soda salt of the acid is dissolved in the blood, which might either be easily decomposed by the carbonic acid (of the tissues), or changed by albumen into an acid salt.

When the author tied the renal arteries he found the same phenomena of precipitation, except in the kidneys, as if he had tied the ureters. This proves that the uric acid excreted by the organism is not generated by the kidneys. The author thinks that the normal blood of birds contains a certain amount of uric acid, and that the kidneys prevent its accumulation in the blood, as their vessels are easy penetrable by the acid. The kidneys play, therefore, only the part of a filter for the uric acid.—*Abstract from Virchow's Archives.—Dr. C. Pawlinoff, of Moscow,* volume 62, 1, 1874. *Western Lancet.*

